# Integrated knowledge translation to strengthen public policy research: a case study from experimental research on income assistance receipt among people who use drugs

**DOI:** 10.1186/s12889-020-10121-9

**Published:** 2021-01-18

**Authors:** Joanna Mendell, Lindsey Richardson

**Affiliations:** 1 British Columbia Centre on Substance Use, Vancouver, British Columbia Canada; 2 Department of Sociology, University of British Columbia, Vancouver, British Columbia Canada

**Keywords:** Integrated knowledge translation, Stakeholder engagement, Evidence-informed policy

## Abstract

**Background:**

Solutions to complex public health issues should be informed by scientific evidence, yet there are important differences between policy and research processes that make this relationship challenging. Integrated knowledge translation (IKT) is a strategy of sustained stakeholder engagement that intends to address barriers to evidence use. We highlight an example of an IKT project alongside a randomized controlled trial of a public policy intervention that tested different disbursement patterns of income assistance among people who use drugs in Vancouver, British Columbia.

**Methods:**

A case study design was used where an IKT strategy led by a knowledge broker embedded within the research team acts as the case. This case study evaluates the process and effectiveness of the integrated knowledge translation project by measuring intermediate outcomes within a Theory of Change created to map pathways to impact. Content analysis was performed using an evaluation template through document review, post-event evaluations, and detailed tracking of media, knowledge translation activities and requests for information.

**Results:**

A host of knowledge translation products synthesized existing research about the harms of synchronized income assistance disbursement and supported stakeholder engagement, facilitating conversation, relationship building and trust with stakeholders. Engagement improved knowledge of the contextual feasibility for system change, and contributed experiential knowledge to study findings. A combination of access to information and stakeholder and media engagement led to increased acknowledgement of the issue by policy makers directly involved in the income assistance system.

**Conclusions:**

This project shows how a multipronged approach to IKT addressed barriers to evidence-informed public policy and successfully contributed to increased public discourse around income assistance policy reform. Additionally, sustained engagement with diverse stakeholders led to improved contextual knowledge and understanding of potential community level impacts that, along with scientific results, improved the evidence available to inform system change. This case study provides insight into the role IKT can play alongside research aimed at public policy improvements.

**Trial registration:**

This IKT project was embedded within the study titled: The impact of Alternative Social Assistance Disbursement on Drug-Related Harm (TASA), known as Cheque Day Study, registered on ClinicalTrials.gov (NCT02457949) May 29, 2015.

**Supplementary Information:**

The online version contains supplementary material available at 10.1186/s12889-020-10121-9.

## Contributions to literature

- Research on knowledge translation predominantly focuses on health care and/or behavioural interventions. This paper expands this literature by focusing on the underexplored area of experimental public policy research.
- This paper adds to the literature on how to evaluate IKT and how to overcome the challenges of evaluating IKT such as data capture from stakeholders and using policy change to measure success of an IKT project.
- This case study examplifies how experiential knowledge and sustained stakeholder engagement can improve public policy-oriented research.

## Background

Relying on traditional avenues of disseminating research (i.e. publications and presentations following the end of a grant) overlooks several issues regarding the use of evidence. Such methods have been criticized for encouraging decision makers to use research without acknowledging the barriers decision makers face in implementing evidence-informed policy making [1]. A strategy shown to be effective in improving the uptake of scientific evidence into public policy development is maintaining ongoing linkages between researchers and stakeholders to improve the evidence that is generated as well as the readiness of the policy environment for the uptake of findings [1–4]. Integrated Knowledge Translation (IKT) is an approach that prioritizes relationships with stakeholders to co-develop and execute research questions [5–8]. In the pursuit of addressing complex social issues, IKT has been encouraged as a way to amplify research impact [5–8]. Utilizing strategies consistent with IKT objectives, knowledge brokers are individuals that work between stakeholder groups, to increase the impact of research evidence [9]. While the role of the knowledge broker will vary between research contexts, having a dedicated knowledge broker as part of a research team is a way to operationalize and formalize IKT within a research project.

While health funders and IKT practitioners promote the use of IKT strategies to influence healthcare policy and professional practice change [10], there are fewer descriptions of the planning, implementation and impacts of IKT processes, especially as they relate to evidence-informed public policy [4]. Additionally, models of stakeholder engagement vary in design and effectiveness depending on the context [11]. To increase the evidence base of IKT planning and implementation processes, we present here a case study of an IKT approach embedded within an experimental study investigating whether alternative income assistance disbursement schedules mitigate payment-coincident drug-related harm. Conducted alongside a randomized control trial of a structural intervention, this case study seeks to describe the planning, implementation and impact of IKT embedded within the study from initiation through to the release of preliminary study findings.

### Integrated knowledge translation and addressing barriers to research utilization

IKT is defined by sustained relationships with stakeholders throughout different stages of research with the intention of improving the strength, relevance, and mutual benefit of a research project [4, 5, 8, 11–13]. While such a process can be described by a range of terms (e.g. knowledge exchange, knowledge mobilization), we used IKT to describe the intentional approach to engaging stakeholders from the beginning to the end of this project. Additionally, as is done elsewhere [14, 15] we distinguish IKT from community-based participatory research (CBPR), which is similar in its underlying strategy to improve research through co-creation of knowledge with stakeholders, but differs in application. A distinction is in how CBPR places control over research within the community and/or knowledge users, and has an embedded commitment to capacity building for community research involvement in addition to the specific goals of the research project [14]. IKT focuses explicitly on expanding the awareness, reach and uptake of research more broadly across sectors [15]. In our research context, where stakeholders spanned several power differentials from community to government, IKT was chosen as an approach to improve the impact of this research.

Reciprocal learning between researchers and stakeholders incorporates a variety of perspectives and experiences and introduces different forms of knowledge when developing the research questions and procedures, considering contextual information, and interpreting results [12, 16]. The intention with IKT is that research questions become more relevant and solutions-based, evidence is more adaptable to decision maker contexts, there is increased trust of researchers and results among knowledge users, and knowledge users become more prepared to use results once they are available [4, 5, 12, 16–18]. While the goals of IKT are well understood, there are research and policy processes that make evidence-informed policy challenging in practice. Among the most problematic barriers to scientific evidence being utilized in policy are research not being directly adaptable to policy contexts or not being available when policy makers need it [19]. The current study outlines an IKT case study whose strategies sought to address these barriers, and contributes to the knowledge base of whether and how such strategies bridge the research-policy divide.

### The research context

This IKT project was embedded within a randomized controlled trial entitled: The impact of alternative social assistance disbursement on drug-related harm, known colloquially in the research site as the Cheque Day Study [20]. In the study context and other jurisdictions, income assistance is commonly distributed once a month to all recipients on the same day. While income assistance critically reduces the harms of poverty [21], the system of synchronized disbursement has been shown to contribute to a monthly cycle of escalations of severe harm coinciding with payments for people who use illicit drugs. This trend has been widely acknowledged in Vancouver, Canada’s Downtown Eastside community, and demonstrated by many years of observational research in Vancouver and across North America [22]. Research points to intensified and riskier drug use following cheque issue as well as increases in related harm such as fatal and non-fatal overdoses, exposure to violence, emergency department use, police service calls, and treatment or health care interruption [20, 23–36].

Repeated calls for changes in the distribution system to disrupt this monthly cycle of harm led to the initiation of the Cheque Day Study. This field experiment examined whether changing the timing and frequency of income assistance payments would mitigate monthly escalations of drug use and subsequent drug-related harms. Housed within the British Columbia Centre on Substance Use and described in detail elsewhere [20] briefly, volunteer participants were recipients of income assistance living in Vancouver, British Columbia, who during screening by the research team reported increases in drug use around payment days. In partnership with a community-located and operated branch of a local Credit Union, the study randomly allocated participants to continue receiving income assistance on government cheque issue days (the study control arm) or one of two intervention arms that differed in either (1) the timing (once a month on a day outside of cheque week) or (2) the timing and frequency of payments (twice a month on days outside of cheque week). The study tests the impacts of changing the income assistance payment schedules as a potential strategy to improve the health and wellbeing for people who use drugs and rely on income assistance as a source of income.

## Methods

Following ethical approval, the Cheque Day Study began recruitment in late 2015. The study had been collecting data for 5 months when a full-time knowledge broker joined the research team to plan and implement an IKT strategy alongside the Cheque Day Study. The IKT project took place over 3 years and sought to amplify the impact the Cheque Day Study would have on mitigating the harm around income assistance payments. The knowledge broker reviewed IKT literature and models, strategically developed an IKT plan, and worked with the research team (the study’s Principal Investigator [LR], Research Coordinator, and Interviewers) to develop KT products, recruit participants, arrange meetings, presentations and exchange events with study stakeholders, and lead consultation for a Community Impact Statement. This Community Impact Statement summarized and highlighted stakeholder concerns and anticipated impacts of changing the way income assistance is disbursed. The knowledge broker kept a detailed impact log that, along with meeting minutes, IKT and media tracking, and an evaluation survey following a key knowledge exchange event (a dedicated Community Forum), acted as the main data for the evaluation of this IKT project. The impact log tracked each activity, its purpose, the related IKT objective, the target audience including characteristics of meeting attendees and stakeholders engaged, distribution, participants/reach, expected outcomes, indicators, challenges/lessons learned, feedback received, and reactions from the research team.

**Fig. 1:**
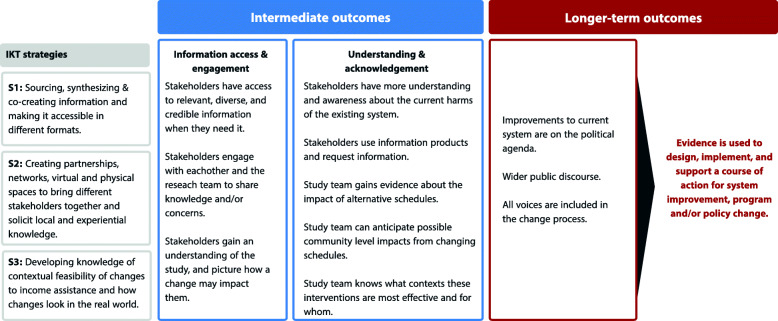
Cheque Day Study theory of change

In keeping with the main purpose of developing Theories of Change, the knowledge broker developed pathways theorized to produce the desired impact (Fig. 1), explicitly outlining its purpose and strategy. The Theory of Change also acted as a tool for evaluating the process of IKT in a way that did not solely focus on the impact of a project, but also on intermediate outcomes theorized to help achieve that impact [37, 38]. The Theory of Change used in this project draws from previous work mapping how information access and stakeholder engagement can lead to social and political change [39] and incorporates strategic IKT areas from the Canadian Institutes of Health Research model of KT [40].

**Fig. 2:**
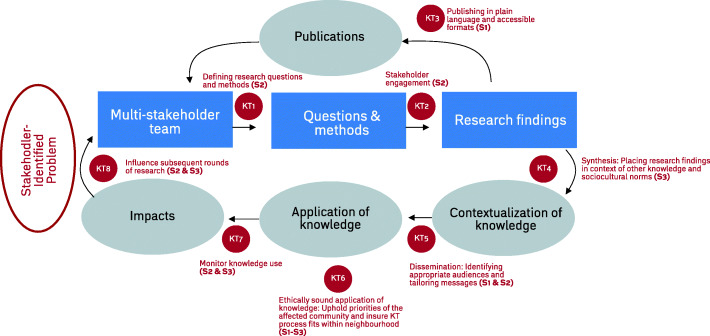
Cheque Day Study integrated knowledge translation model

Working backwards from aspirational high-level outcomes, measurable intermediate outcomes and assumptions were outlined in the Theory of Change to conceptualize how that impact would be achieved. Three main areas of IKT strategy were emphasized, drawing on IKT literature that identifies strategies that connect knowledge-based objectives with research processes: 1) Sourcing, synthesizing & co-creating information and making it accessible in different formats [5, 8, 39]; 2) Creating partnerships, networks as well as virtual and physical spaces to bring different stakeholders together and solicit local and experiential knowledge [5, 8, 11–13, 39]; and 3) Developing knowledge of the contextual feasibility of changes to income assistance disbursement and how changes might be implemented in a real-world context [12, 16, 41]. The CIHR model of IKT and Knowledge to Action Cycle [40] was then adapted into a study-specific model for IKT to highlight opportunities for IKT activities across the research cycle. Activities to propel the underlying strategies of Theory of Change were identified and added to this IKT model, as were the corresponding strategies from the Theory of change (S1-S3) (Fig. 2).

### Data sources

To describe this IKT case, data were collected through document review, an evaluation survey following the Community Forum (Supplemental Table 1) and a detailed tracking of media, IKT activities and stakeholder requests for information or study results. An impact log was kept detailing each activity, reach (e.g., event attendance, number of KT products disseminated) and indicators of impact (e.g. knowledge users requesting study findings, policy makers publicly discussing the study or its preliminary results). Minutes of meetings with stakeholders were also reviewed, as were media stories. Following knowledge exchange events, we asked participants to share their experience through a ‘dotmocracy’ style poster where people were asked to place stickers on a poster to answer evaluation questions such as “*Were you able to share your opinion?*” or “*Did you learn something about the research today?*”

### Analysis

Content analysis was performed by the first author to identify how IKT activities contributed to each of our three IKT strategies and indicators of outcomes outlined in our Theory of Change. An initial coding template was developed based on predetermined evaluation questions designed to test how well intermediate outcomes were reached (Table 1).

**Table 1:** Example of evaluation questions in coding template

Examples of evaluation questions and sub questions		Indicators, *recorded at evaluation checkpoints 6 months/1 year/2 years*
Question 1	How well is the study exchanging knowledge and soliciting feedback from stakeholders?	
Sub questions	Is the study effectively creating lines of communication with stakeholders? Does the study team create an environment for reciprocal learning?	• # of KT events • # of people indicating they learned something at a KT event • reflection from research team
	Is the study effectively synthesizing information about existing harms of cheque day?	• # of different KT products produced • Feedback on KT products from stakeholders
Question 2	How well is the study developing knowledge of the contextual feasibility of ‘solutions’?	
Sub questions	What information is the study collecting about the wider context that affects/is affected by a change in income assistance?	• # of meetings with stakeholders • # of stakeholder groups contributing knowledge
	Is the study considering other sources of knowledge? (i.e. experiential)	• inclusion of experiential knowledge in KT products

Consistent with developmental evaluation principles, where questions are asked continuously throughout the lifespan of a project in order to improve and adapt processes in real time [42], coding was ongoing as research progressed, with defined check in points at 6 months, 1 year, and 2 years. This ongoing approach to evaluation was chosen to help ensure we were meeting IKT goals, nd knowledge needs of stakeholders in a unique and complex context for IKT. Analysis was performed through document review of meeting minutes, the Knowledge Broker Impact Log, media stories and the Community Forum post-event evaluation survey. In reviewing each of these data sources at the 6 month, 1 year, and 2 year check points, data was input into the coding template, at which point data were summarized in an interim evaluation report identifying ‘successes’, ‘gaps identified’, ‘priorities moving forward’, and ‘next steps’.

## Results

### Evaluation of IKT activities

Between the fall of 2013 and Spring 2019, over 600 individuals were consulted as part of the IKT project. This number is an approximation as tracking the exact number of participants was difficult for some IKT events (e.g. community event-based outreach such as a booth at a Health Fair). A total of 67 organizations were consulted with an additional 44 directly contributing to study recruitment. KT products included: (1) plain language summaries; (2) infographics and community-tailored research postcards summarizing pre-existing research about the harms of synchronized income assistance disbursement; (3) briefing notes; (4) technical reports; (5) research summary reports as results became available; and (6) the Community Impact Statement. Engagement with study stakeholders included presentations with clinical and community service providers, regular communication and meetings with policy makers, community events with people in the Downtown Eastside (including a Community Forum hosting 36 individuals), and eight other community engagement events. Additionally, we hosted two forums with first responders including police, firefighters and paramedics. These engagement events contributed greatly to our third goal of developing knowledge about the contextual feasibility of changes to the income assistance schedule, and provided the basis for the Community Impact Statement. A summary of all IKT activities is provided in Table 2 and their contribution to meeting the intermediate outcomes outlined in the Theory of Change, 1) Information access, 2) Engagement, and 3) Understanding and acknowledgement are discussed in detail below.

**Table 2:** Reach metrics by IKT strategic area

Quantity	Description of IKT product or activity
A. Synthesis and dissemination	
14	KT products summarizing existing research on harms coinciding with synchronized income assistance disbursement (e.g. plain language summaries, research summary postcards and infographics)
2	Graphic recording posters creating during the Community Forum
49	Media stories citing research/interviews with study team
8	Media stories related to the issue of cheque day
1	Study webpage hosting KT products
2	KT products summarizing interim results for provincial policy makers (briefing note and technical report)
4	KT products summarizing initial results for different audiences (briefing note, technical report, one-page summary of results, and an 8-page summary of initial analyses)
7	Presentations of initial results (1 people with lived experience(s), 2 academic, 1 policy makers, 1 community, 1 mixed audience including researchers, policy makers, service providers etc., and 1 presentation to Vancouver City Council)
B. Networking, relationship building & communication	
7	Key relationships established prior to study initiation) with representatives from The BC Ministry of Social Development and Poverty Reduction, The BC Ministry of Health, Vancouver Coastal Health, City of Vancouver, Vancouver Police Department, Providence Health Care, PHS Community Services Society, VanCity Savings and Credit Union, Vancouver Area Network of Drug Users, and the Western Aboriginal Harm Reduction Society
22	Presentations/consultations with clinical and community service providers
1	Community Forum with 36 individuals representing 25 organizations (community members and community service providers)
8	Community engagement events (6 organized by us, 2 we attended)
7	Other stakeholder engagement meetings (research groups, community networks)
9	Meetings with policy makers
2	First responder forums attended by 25 police officers, fire fighters and paramedics
600	Individuals involved in knowledge exchange events
67	Organizations consulted
44	Other organizations involved with recruitment
2	Newsletters disseminated to 107 stakeholders
C. Developing knowledge of contextual feasibility	
1	Provincial survey about experiences with cheque day and potential impacts of changes the system with 39 respondents from 8 communities across BC
1	Video that shared experiential knowledge in the form of interviews with people affected by Cheque Day
1	Community Impact Statement (report summarizing all consultation work)

### Information access

Sourcing, synthesizing, and summarizing existing research and making it available to stakeholders was a valuable first step in this project. Most IKT activities required providing background to explain the problem and why the research was being done. Summarizing and synthesizing this work into stakeholder-appropriate materials facilitated conversation with stakeholders, and in some cases served as a pretext for engaging with stakeholders. This was an important part of improving understanding and awareness. Additionally, requests were commonly made from different stakeholders (e.g. at community events) for information about where alternative income assistance schedules had been tried. Knowing where the gaps in information were was important to increase understanding and awareness, develop knowledge of why the study was needed as well as improve trust and relationships between stakeholders and the research team.

Stakeholders had access to a variety of KT products accessible from the study website with varying levels of detail to allow them to choose the level of information they wanted or needed. Another strategy was to use visually engaging media that were informative and providing information alongside something useable. For example, research summary postcards displayed an original and picturesque photograph taken in the Downtown Eastside neighbourhood that could be used as artwork on one side and included information regarding a published research study on the back. Feedback from the community about the design of these KT products was overwhelmingly positive.

### Engagement

Engagement with stakeholders began before study initiation with representatives from provincial and municipal government and health authorities, community organizations, service providers and people with lived experience(s). This early engagement informed the study protocol and research procedures and served to raise awareness about the issue and the study. As research progressed, the study team maintained lines of communication with these and additional stakeholders through email or phone call updates, presentations, meetings or study newsletters, with tailored strategies for different stakeholder groups. For example, email or phone call updates were the most appropriate for some key stakeholders such as those in policy and/or leadership positions, while for local residents and people with lived experience(s) presentations, community events, and newsletters were better suited to community needs.

#### Networking and accessing diverse perspectives

As stakeholder engagement continued, a number of key partnerships developed with established coalitions and organizations that do outreach in the community as part of their organizational mandates. Connecting with these organizers in the study context was instrumental in developing a broader network, securing access to a wider range of organizations and connecting with people that had not been interested or willing to speak with us prior to being introduced through these organizations. Conversations with harder to reach groups were essential in our efforts to collect contextual information as they provided different and contrasting perspectives than those already supporting the changes being evaluated by the Cheque Day Study. In addition to refining investigators’ understanding of their reticence for change, the varying perspectives collected from stakeholders were highlighted in a Community Impact Statement. This Community Impact Statement documented diverse perspectives in a balanced way, intending to profile the importance of considering different viewpoints by policy makers when designing policy changes that will differentially impact many stakeholders.

Access to diverse perspectives ensured that these could be included and centered on an ongoing basis, which we believe reduced community stakeholder perceptions of bias, this was certainly salient at The Community Forum. This forum was held at a university-affiliated organization in the Downtown Eastside with a central storefront location and attended by 36 people who live and/or work in the neighbourhood, with 25 organizations represented at this forum. Convening of people from different organizations alongside neighbourhood residents provided a forum to hear each other’s concerns, exchange ideas, and ask the research team questions. Several people indicated that having the chance to discuss with other organizations helped them better understand the context and perspectives outside their own experience. In other community meetings community members voiced how it felt good that their opinions seemed to matter. One example is from a resident at a local single room occupancy hotel, whose feedback stated: “I am glad to see my insight be valid & validated, Thank you”.

#### Policy engagement

As income assistance is managed through the BC Ministry of Social Development and Poverty Reduction, and the health impacts of drug-related harm were of core concern to the Ministry of Health and Ministry of Mental Health and Addictions, provincial policy makers were a priority stakeholder group. Despite interest in reducing the health harms from substance use from officials in the Ministry of Health, at study initiation policy makers directly involved in income assistance policy identified that changing the schedule of income assistance disbursement was not a priority. Consistent with the Theory of Change and its constituent strategies, communication began prior to study initiation and was sustained throughout the study with the intention to prime the political environment for the uptake of study results. Initial indicators of successful engagement with policy makers came from media statements by government officials. In March 2018, the Minister of Social Development and Poverty Reduction said in a media interview: “We know there could be unintended consequences of making these changes, and we’re very much interested in determining what those implications might be. That’s why we’re supporting the research that’s being done by the BC Centre on Substance [Use]. They’ve been looking exactly at this cheque day issue, and what the impacts are. And I’m hoping that we’re going to see some results from that in the coming months” [43]. Additionally, in April 2019 the Minister of Mental Health and Addictions provided the following statement in a media interview in response to an opposition politician tabling a private members bill requesting that a revision to the income assistance disbursement schedule be explored: “We will see what [the BC Centre on Substance Use] has to say and we will take action if it means people making people safer” [44]. Additional discussions with senior policy and decision makers indicate a high level of interest in the results from this research, suggesting the importance of early and consistent engagement. Outside of provincial ministries, senior officials within health authorities have also been engaging with study results, indicating their interest in examining potential policy reforms.

#### Engagement with Cheque day study results

As preliminary results from the scientific study were complex, identifying signals for both benefit and increased harm from a revised schedule [45, 46], engagement in the weeks leading up to preliminary results being available involved the development of a coordinated strategic launch to the scientific community, policy and community stakeholders, and the media to improve the likelihood that results would be interpreted in a balanced way, avoiding oversimplification or sensationalized reporting. Ahead of the public release of preliminary findings, the research team consulted people with lived experience(s) around findings and recommendations, provided an embargoed news release and accompanying media interviews, and communicated with policy makers in government, providing a confidential technical report and policy brief. The messaging around the complexity of results and nuanced recommendations were successfully communicated as evidenced by the narratives surrounding the release of findings including media stories as well as comments from government officials and community stakeholders.

Another indicator of engagement with the research was the more than 80 requests for results that have come from several stakeholder groups, including provincial ministries, health authorities, public health organizations, first responders, media, community organizations, service providers, other researchers, and private individuals interested in results, including a keynote presentation to policy makers, academics, and first responders at summit coinciding with the release of results.

### Understanding and acknowledgement

One of the most significant outcomes of this IKT project was the reciprocal learning that study team was able to do with stakeholders (community members, clinical and community service providers, first responders, policy makers at multiple levels of government) and the contextual knowledge this work was able to provide. While at the outset of the project, the main IKT goal was to help research results have impact, as we learned more, and witnessed variation in experiences for participants, we realized the potential importance of the IKT in terms of developing a more fulsome understanding of the study context, the value of listening to and incorporating concerns in the community into the broader study process and anticipating potential impacts of a change to the income assistance distribution schedule.

#### The community impact statement

Given the range and heterogeneity in concerns expressed by stakeholders about a change in the income assistance disbursement schedule, our IKT goals evolved to prioritize highlighting experiential knowledge in the community of relevance for policy makers and service providers should reform be considered. This was undertaken through the development of a Community Impact Statement, a report summarizing perspectives gathered from stakeholders including recipients of income assistance, people who use drugs, people who provide support services, first responders and policy makers at various levels of government. As heard during a community meeting, speaking with people who might not want or be able to participate in the study was an important way to expand our knowledge about the context, and potential implications of policy reform. One community member articulated, “Even if the study is voluntary for people who want to make this change, a change may not be voluntary, so you need to think about who may be affected by a change and speak to them, whether or not they are wanting to take part in the study”. The Community Impact Statement served as an indicator of the reciprocal learning and a centering of diverse viewpoints. It increased understanding that the research team drew significantly on from consultations in their work and was a tool to increase understanding and awareness for those engaging with results. This Community Impact Statement was presented alongside scientific results and was referred to both by the media and by policy makers.

#### Public acknowledgement of the issue

In the lead up to the release of research findings in spring 2019, policy makers from three provincial ministries requested study results. The research team met with senior policy makers to present initial findings to those who showed interest in study results. Key indicators that income assistance disbursement schedule reform is being considered in the public domain include a Private Member’s Bill being submitted by the opposition government in the Legislative Assembly of British Columbia calling for changes to the income assistance schedule to be considered [47], and Vancouver City Council passing a motion to support changes at the provincial level [48], during the deliberations for which the PI was invited as a speaker. Political activity in support of income assistance schedule reform reflects a significant change in acknowledgement of the issue by those responsible for income assistance policy or in positions to advocate for policy reform at senior levels of government. Whether there will be change and how changes would be implemented are yet to be seen, however these results are indicative of success with the intermediate outcomes of engagement, increased awareness, and acknowledgement of the problem.

## Discussion

Solutions to complex public health issues, like the monthly harm of synchronized income assistance, should be informed by the best possible evidence, but there are barriers that often prevent the uptake of available evidence. These include, but aren’t limited to, the timing of research not matching up with windows for policy change, research not being directly relevant or adaptable to policy context, the absence of personal contact between researchers and policymakers, research evidence conflicting with policy or political agendas, and insufficient evidence from a practical implementation perspective [11, 49]. In the case of the Cheque Day study, while multiple studies had previously called for a change to the distribution schedule of income assistance, discussion of that change was largely absent from the public sphere, and no scientific evidence had explored the practicalities and potential impacts of implementing such a change. To supplement the Cheque Day Study, which to our knowledge was the first study to experimentally test alternatives to synchronized income assistance payments [20], an IKT Theory of Change was developed aimed at addressing barriers to the use of evidence. This Theory of Change tested the hypotheses that sustained engagement and access to accessible information would improve understanding and awareness of the research-related issues among knowledge users, in turn leading to increased buy-in and strengthened potential that research results would support policy or programmatic change. Additionally, it anticipated that meaningful engagement with stakeholders would improve the quality, relevance and usability of research evidence by improving the understanding of the context and potential impacts of a change among researchers.

### Beyond policy impact

The Cheque Day Study IKT project took on greater importance as the study progressed than initially conceptualized. In the best-case scenario where a study is able to provide a clear recommendation for action, IKT intends to amplify the impact of research evidence through mechanisms like co-creation of knowledge, priming the policy environment for the uptake of findings, and improving trust with stakeholders, including policy makers and community members [1, 5, 8, 11–13, 16]. In more complex cases, this work can become even more important to add depth of and nuance to stakeholder and public understanding. In the current case, consideration of the complexity of results was and continues to be needed from stakeholders to determine an ethical, evidence-based path for reform. The IKT activities undertaken alongside the research critically provided contextual understanding, legitimacy among stakeholders and insight into the potential impacts of policy change. As such, IKT can expand the scope and reach of a project, ensuring appropriate impact within and beyond the original project goals.

In the current study context it was important to listen to a broad range of stakeholders and revisit and revise the Theory of Change as more information became available. It was also important to be flexible and adapt planning where originally conceptualized activities would not have effectively achieved high-level impacts as the project, research environment and policy context evolved. For example, the Community Impact Statement was not part of our initial workplan, but as stakeholders expressed heterogeneous and often contradictory concerns about income assistance system reform, an important part of providing the best possible evidence to relevant stakeholders required the inclusion of this information. The Community Impact Statement directly reflects the reciprocal learning that occurred during consultation and addressed the intermediate outcomes goals of understanding the context for implementing change as well as being able to anticipate community-level impacts from change.

### Importance of experiential knowledge

IKT research, as with other participatory research methods, highlights the value of including other forms of knowledge together with scientific evidence and academic expertise [1, 3]. The importance of experiential knowledge became increasingly evident as this IKT project progressed. It was important to reflect that study participants had varying experiences of the intervention and people consulted during the IKT engagement process anticipated impacts outside of those witnessed in the study population. This also promoted understandings that some of the potential impacts would be research study-specific: the study changed the schedule for only a small proportion of those receiving income assistance in the community. Stakeholders discussed potential impacts of widespread system change if everyone in the community were paid on different days. As participation in the study was voluntary, observations within the study sample may change if such reforms are brought to scale. As such, consulting with people who were not interested or eligible to participate in the study provided more understanding about what broad change might look like in communities. This heterogeneity in the community perspectives, alongside complex research findings supported recommendations to government that focused on developing the capacity to adapt individual payment schedules to recipient needs. The experiential knowledge collected alongside the study will provide invaluable information regarding next steps in any potential reform process.

### Limitations

A limitation of this case study was the restricted amount of data we were able to collect about IKT activities from stakeholders. In projects like this, where we are asking for people’s time to engage with our research and to provide their expertise, it is often inappropriate to subsequently ask them to spend time answering questions regarding their engagement with us, and even less so to ask for before-and-after data on either side of an IKT activity to test changes in awareness or understanding. This was particularly true for two of our key stakeholder groups, government officials and community members in the Downtown Eastside of Vancouver, for different, but important reasons. Briefings with policy makers involved us requesting time from government officials to engage with us about the research, with the limited amount of time awarded for these conversations, our time was devoted to discussing the substantive issue at hand rather than KT processes. In such instances it may not be appropriate after a meeting for us to ask for feedback regarding the influence the meeting had, and requesting additional time in this way could negatively impacted future requests for meetings. Additionally, we wanted to be cognisant of the response burden on a heavily researched population in the Downtown Eastside of Vancouver [47]. We thought carefully about time we were asking people to invest, and of how to ensure that this time was meaningfully contributing to improvements in research***.*** It was therefore not part of our evaluation design to conduct such interviews with stakeholders to track how our IKT work influenced understanding, awareness, political will, or whether and how it met stakeholder information needs. In the case of the Community Forum it was possible to conduct an evaluation survey to elicit some of this information. Another strategy we used to elicit feedback after community engagement events was “dotmocracy”, but such attempts had very low participation, potentially pointing to low interest to provide additional self-reflexive feedback in IKT processes in a heavily researched community. Extracting data from stakeholders is particularly challenging in a project that prioritized soliciting experiential knowledge from many stakeholder groups to enrich the contextual information of the research. Instead of having a small group of deeply involved stakeholders, we had a large group of stakeholders who engaged in a less consistent way.

Additionally, this case study relied on evaluating intermediate outcomes that the Theory of Change hypothesized could influence a policy change rather than directly measuring policy change. While evaluating long-term change ideally describes how IKT can amplify research impact, this type of intermediate reporting can help deepen understanding of the pathways to impact and is an important but often neglected part of the evidence base for this type of research activity. Measuring the impact of IKT through policy change is unrealistic in many cases, and may miss the wider impact IKT can have on a research project or public policy area. Kothari and Wathen (2013) discuss, the “positivity bias” where researchers assume that findings of a study will contribute to positive change. Due to this bias, researchers and stakeholders alike will go into a project assuming that change will be warranted from findings of the study. In many cases a single study will not provide the evidence necessary to elicit such change [1] and it if does, it is difficult to attribute causation to a specific study [50]. Therefore, using policy change to measure success of IKT may unfairly evaluate a project as unsuccessful. Measuring intermediate outcomes may help to describe benefits of IKT independently of policy change. The challenges in measuring the impact of IKT may contribute to the lack of reported cases of IKT. We chose to report on our project in the absence of (or advance of) any policy change to contribute to the evidence base for the impact of IKT on public policy research, and help inform other projects implementing similar IKT interventions.

Lastly, this IKT project was led by a dedicated knowledge broker [JM] who over the course of three-years worked full time to plan and execute the activities described here. Even whilst having a full-time knowledge broker, this IKT project demanded a substantial amount of time from the study’s PI and Research Coordinator. Having a dedicated position for IKT is not common, and as such, replicating a similar IKT project could be difficult for a research team considering the time commitment required to undertake this work.

## Conclusion

This case report details an IKT project nested within a policy-relevant experimental study, and outlines how sustained stakeholder engagement impacted the depth of understanding and acknowledgement of the issues surrounding synchronized income assistance disbursement. However, a number of factors make it difficult to draw conclusions about the direct pathway between efforts and outcomes from in this project. The difficulty with data capture from knowledge users and the inappropriateness of before-after testing in our context made it challenging to assign causality to individual knowledge exchange activities in relation to our stated objectives. However, we are able to conclude that the combination of activities undertaken alongside the Cheque Day Study, in conjunction with the fact that this study sought to resolve a salient social and health issue, contributed to a number of outcomes of importance to reform efforts. For example, the IKT activities in this report raised considerable awareness and attention: the provincial government admitted the current system is flawed [43, 44]; we received requests for scientific results from over 80 individuals and organizations including the provincial ministry that manages the income assistance system; there was widespread media engagement and coverage; there has been considerable political activity and public references to the study by senior elected officials; the study captured invaluable experience within the community in a Community Impact Statement that accompanied research results and helped craft nuanced recommendations for policy change.

The IKT alongside the Cheque Day Study helped prime the research context for study results to support the development of a more public-health promoting income assistance disbursement system. We anticipate that evidence will be used by policy makers and service providers to improve the current system through changes within the Ministry of Social Development and Poverty Reduction, programming through community organizations to support people around income assistance payments, and the possibility to develop innovative private solutions to support public health-promoting income assistance disbursement strategies.

Our experience with this IKT project supports the necessity for multipronged approaches that address barriers to evidence usage in specific research contexts. As such, to maximize the policy and community impacts of research requires more than generalized end of project KT efforts that expect policy makers to simply adopt evidence-based recommendations. Synthesizing and summarizing related research was important to outline the impetus for the study, and helped with stakeholder engagement and relationship building. Building relationships and meaningful consultation with stakeholders added the value of experiential knowledge, improved the depth of understanding that the research team was able to incorporate in their efforts and provided invaluable contextual knowledge for policy reform recommendations and efforts. Sustained relationships with a wide array of stakeholders, in addition to providing invaluable experiential knowledge, led to substantial engagement with results and the issue being discussed in community, among service providers, in the general media as well as in municipal and provincial governments, indicating engagement, awareness, and acknowledgement of the issues associated with synchronized income assistance disbursement. As demonstrated by this project, IKT practice has the potential to improve the quality and usability of research by including experiential knowledge, influence public acknowledgement of an issue, and amplify the impact of public-policy focused research.

## Supplementary Information

**Additional file 1: Supplemental Table 1.** Community forum evaluation survey

## Data Availability

The datasets used and analyzed for the current study will be available from the corresponding author upon reasonable request.

## Abbreviations

IKT: Integrated Knowledge Translation
KT: Knowledge translation
TASA: The impact of Alternative Social Assistance
