# Supplementary material for: Integrated knowledge translation to strengthen public policy research: a case study from experimental research on income assistance receipt among people who use drugs
Source: BMC Public Health. 2021 Jan 18;21:153. doi: 10.1186/s12889-020-10121-9 (PMC7814536; doi:10.1186/s12889-020-10121-9)
Supplement: Supplementary file 1 — Additional file 1: Supplemental Table 1. Community forum evaluation survey [file 12889_2020_10121_MOESM1_ESM.docx]

# **Supplemental Table 1. Community forum evaluation survey**

| **Question** | **Response** |
| --- | --- |
| Did the event meet your expectations? | - much better than expected - better than expected - about what I expected - worse than expected - much worse than expected |
| Was the content level of the presentations understandable? | - very easy to understand - easy to understand - neutral - difficult to understand - very difficult to understand |
| Did you think this was a good format to share your thoughts, ideas, and experience? | - very much so - somewhat - neutral - not really - not at all |
| Overall, what did you find most useful about the event? | Open ended |
| What is one thing you would change? | Open ended |
| Additional comments? | Open ended |
| We will be continuing to gather feedback about how changing income assistance payment schedules might impact community. If you would like to arrange a time to speak further, or have us visit your organization to discuss the study, please let us know and provide your contact information. | Open ended |
